# A Comparative Transcriptomic Analysis of Uveal Melanoma and Normal Uveal Melanocyte

**DOI:** 10.1371/journal.pone.0016516

**Published:** 2011-01-28

**Authors:** Jianhong An, Haolei Wan, Xiangtian Zhou, Dan-Ning Hu, Ledan Wang, Lili Hao, Dongsheng Yan, Fanjun Shi, Zhonglou Zhou, Jiao Wang, Songnian Hu, Jun Yu, Jia Qu

**Affiliations:** 1 School of Optometry and Ophthalmology and Eye Hospital, Wenzhou Medical College, Wenzhou, China; 2 State Key Laboratory Cultivation Base and Key Laboratory of Vision Science, Ministry of Health and Zhejiang Provincial Key Laboratory of Ophthalmology and Optometry, Wenzhou, China; 3 CAS Key Laboratory of Genome Sciences and Information, Beijing Institute of Genomics, Chinese Academy of Sciences, Beijing, China; 4 Tissue Culture Center, The New York Eye and Ear Infirmary, New York Medical College, New York, New York, United States of America; 5 Graduate University of Chinese Academy of Sciences, Beijing, China; Memorial Sloan Kettering Cancer Center, United States of America

## Abstract

**Background:**

Uveal melanoma is the most common primary intraocular tumor in adults in western countries. It is associated with very severe visual morbidity and may lead to distant metastases even after successful treatment of the primary tumor. In order to gain better insight into molecular mechanisms related to tumorigenesis and metastasis of uveal melanoma, we used next-generation sequencing technology (SOLiD, Life Technologies) to acquire global transcriptome alteration between posterior uveal melanoma cells and normal uveal melanocyte.

**Results:**

From mRNAs of the cultured uveal melanoma cells and normal uveal melanocytes, we annotated more than 3.7×10^7^ and 2.7×10^7^ sequencing tags based on human Ensembl databases, respectively. For detailed analysis, we chose 5155 well-annotated genes mainly involved in the MAPK signaling pathway, cell cycle, cell adhesion junction, apoptosis, and P53 signaling pathways as well as melanogenesis. In an effort to confirm the authenticity of our sequencing results, we validated twenty-one identically differentially expressed genes by using quantitative real time PCR from cultured cell lines of other posterior uveal melanoma cells and normal uveal melanocytes.

**Conclusion:**

We have identified a large number of potentially interesting genes for biological investigation of uveal melanoma. The expression profiling also provides useful resources for other functional genomic and transcriptome studies. These 21 potential genes could discriminate between uveal melanoma cells and normal uveal melanocyte, which may be indicative of tumorigenesis process. Our results further suggest that high-throughput sequencing technology provides a powerful tool to study mechanisms of tumogenesis in the molecular level.

## Introduction

Uveal melanoma (UM) is an intraocular malignant tumor occurring mainly in adult Caucasian and originates from melanocytes of the choroid, iris, and ciliary body [1], [2]. Most of UMs (95%) are posterior UM (locating in the ciliary body and choroid). Over a 25-year period from 1973 to 1997, incidence of UM in the United State has been determined to be 4.3 cases per million people per year, which is similar to the report from European countries [3]. Though UM is relatively rare compared with other malignant tumors, it contributes to a large proportion of death rates and leads to distant metastases even after successful treatment of the local tumors [4]. Effective prevention and treatment of metastatic UM remain elusive until now and overall survival rate of patients with UM has not decreased in the past decades [5], [6].

Early studies mainly focused on the cytogenetic variation and chromosomal alterations of UM. The ploidy states of UM cells indicate that aneuploidy is associated with epithelioid cell type [7], [8]. Aneuploidy of UM is a signal for poor clinical prognosis with an increased rate of mortality [9], [10], [11]. Subsequent researchers found that loss of chromosome 3 (monosomy 3) and gain of chromosome 8q were associated significantly with high death rates of UM [12] and the loss of 6p was a specific character of primary UM metastases [13], [14].

In the past decades, various gene mutations have been reported in cutaneous melanoma, including BRAF, NRAS, p16 (CDKN2A), p53, PTEN, etc [15]. None of these gene mutations have been detected in posterior UM [16]. Recently, it has been reported that mutation of GNAQ gene (gene encodes heterotrimetric G protein alpha-subunit) occurred in approximate 50% of UM patients, but not in cutaneous melanoma [17].

In the stage of molecular studies, oncogenesis of UM is considered to be a multistep, complicated process due to excessive acquired or inherited genetic alterations that result in abnormal regulation of multiple key cellular pathways [18], [19]. Many functional alternations have been found in related pathways, including cell cycle [20], apoptosis [21] and P53 pathway [22], [23]. In the meantime, several regulation factors have been found and applied as the diagnostic markers in clinical and histopathologic examinations, such as P53, Ki-67, Laminin Receptor 1 (LAMR1), Endothelin 2 (ET2), Von Hippel Lindau Binding Protein 1 (VBP1) and Cullin 2 (CUL2) [24], [25], [26].

To further survey molecular mechanism of UM and to understand the oncogenesis of UM, we used the recently-developed RNA-seq method to identify and quantify different gene expression between UM cells and normal uveal melanocyte (NUM) so that we could interrogate the dynamic variation of UM transcriptome [27]. Our data demonstrated a greatly transformation of transcriptional activity in the whole genome scale of UM as compared with NUM, leading directly to the up-regulation and down-regulation of thousands of genes involved in various important functional pathways. The most apparent disturbances of multiple functional pathways other than the cell cycle, apoptosis, and P53 signal pathways include mitogen-activated protein kinase (MAPK) signaling pathway, cell adhesion junction, and melanogenesis pathways. In an effort to validate the authority of SOLiD data, quantitative real-time PCR (qRT-PCR) analysis on several differently expressed genes was used in cell lines from distinct UM and NUM specimens.

## Materials and Methods

### Source of the samples

The human uveal melanoma cell line M23 were isolated from Caucasian patients with primary choroid melanoma using methods described previously by us [28], grown in Dulbecco Modified Eagle Medium (DMEM; Invitrogen, Carlsbad, CA) supplemented with 10% fetal Bovine Serum (FBS; Invitrogen-Gibco, Carlsbad, CA) and incubated at 37°C in a humid incubator containing 5% CO_2._ The human melanocyte cell line UM-U-95 was obtained from the choroid of healthy Caucasian donor eyes using the methods we described previously [29]. All studies and procedures involving human tissue were approved by the Wenzhou Medical College Institutional Review Board. Patient samples involved in this study were used in accordance with the tenets of the Declaration of Helsinki adopted by the World Medical Association, 1964. Written consent was obtained from every patient after being fully informed of the purpose of the study.

### RNA extraction and library construction

About 5–10×10^6^ cultured cells were used for the isolation of total RNA by Trizol Reagent (Invitrogen, Carlsbad, CA) at no less than 1 µg. High-quality mRNA of each samples were extracted with the Oligotex mRNA Spin-Column Kit, following the protocols of the manufacturer (QIAGEN, Valencia, CA). After the fragmentation of mRNA, 50–150 nt cleanup fraction were collected for hybridization and reverse transcription. Then, the cDNA library was amplified, cleaned with Qiagen MinElute PCR purification Kit (QIAGEN, Valencia, CA) and purified on a native 6% polyacrylamide gel. Different PCR primers with corresponding barcodes were connected with cDNAs for the PCR amplification. About 140–200 bp cDNA (corresponds to about 50–110 nt mRNA) were excised by PAGE and then concentrated into 10 µl after purification. Library examination was required and the results of more than 80% proportion mRNA in each library could be used for subsequent SOLiD sequencing (**Table S1**).

### SOLiD sequencing and alignment strategy

Both libraries were sequenced using SOLiD (Applied Biosystems, Foster City, CA) sequencing technology. Over 500 pg cDNA of each library was driven onto 1-µm-diameter beads using emulsion PCR. The corresponding barcode primers representing different samples enable us to run two completely independent samples in a single run. We sequenced more than 4×10^7^ tags on a SOLiD sequencing, approximately 70% of which were high-quality sequence tags 35 nt in length.

The SOLiD alignment software (Applied Biosystems, Foster City, CA) translates the reference sequence to di-base encoding (“color-space”) and aligns the reads in color space, which avoids leading to the scale-up mismatches. The software guarantees finding all alignments between the reads and the reference sequences with up to M mismatches (a user-specified parameter).

### Construction of reference database and annotation

Reference database were made respectively by ribosomal RNA and human chromosome database. Since parts of mature mRNAs originated from diverse exons owing to alternative splicing, exon junction database was created to annotate these categories of transcripts. The exon junction database was composed by the randomly combination of 34 bp sequences near the exonic junction points. Low-quality reads (with the average quality value below 8) were filtered and the remaining reads were used for further mapping steps.

The 35 bp data were firstly matched with ribosomal RNA database to wipe off the disturbance of remnants of rRNA. The left data were then aligned with Human genome and exon junction database for annotation successively, with the allowance of three mismatches. The last 5 bp were cut off from unmapped tags for the next matching with chromosome and exon junction database (allowing two mismatches) to increase annotation rate of raw data. Similar 25 bp matching with was made after the 30 bp alignment as well. The two libraries tags were mapped to the Human genome (Ensembl database) allowing up to three mismatches for each read. Finally, after these three steps, tags with matching information were collected for subsequent biological analysis.

### Biological analysis methods

Gene expression profiling analysis was based on the number of tags matching with exon regions. Genes matched with more than one tag on exon regions were considered to be expressed in our statistical method. Also, RPKM (reads per kilobase of exon model per million mapped reads) were used to evaluate the expressed value and quantify transcript levels [30].

Functional classes were assigned according to GO (Gene Ontology) mapping provided by the Ensembl database. DEGseq program was used to make statistical analysis for the differentiated gene expression between the two samples [31]. Genes with p-value less than 0.01 were considered to be distinctly expressed. KEGG analysis was based on the comparative results between our mapping genes and the updated KEGG database [32], [33]. GenMAPP 2.0 and PathVisio were respectively used to evaluate differentiated gene expression in a variety of biological pathways [34], [35].

### Quantitative real-time PCR analysis

The samples for qRT-PCR were derived from the human posterior UM cell lines M17, M21, M23, and SP6.5 (provided by Guy Pelletier, Research Center of Immunology, Quebec, Canada), which were isolated from Caucasian patients with primary choroid melanomas and grown in the same medium using above method described previously [36], [37]. The human melanocyte cell lines UM-U-90, UM-U-94, UM-U-95, UM-U-97, UM-U-106 were isolated and cultured as previously described as well [28], [29]. All studies and procedures involving human tissue were approved by the Wenzhou Medical College Institutional Review Board. Patient samples involved in this study were used in accordance with the tenets of the Declaration of Helsinki (adopted by the World Medical Association, 1964).

Gene sequences were obtained from the National Center for Biotechnology Information (NCBI) with the primer design detailed in **Table S2**. For qRT-PCR analysis, total RNA was extracted based on the Trizol protocol (Invitrogen, Carlsbad, CA), treated with DNAase I(Promega, Madison, WI), and reverse-transcribed to cDNA (random priming) by using a standard protocol (SuperScript II reverse-transcriptase, Invitrogen, Carlsbad, CA). The PCRs were performed in an Applied Biosystems 7500 quantitative Real-Time PCR System using 2× SYBR® Green PCR Master Mix (Applied Biosystems, Foster City, CA). The initial denaturation reaction conditions for qRT-PCR were 50°C for 2 minutes and 95°C for 10 minutes, followed by 40 amplification cycles at 95°C for 15 s, 60°C for 60 s, and 68°C for 40 s.

Each sample was analyzed in duplicate with the volume of a single reaction added up to 15 µl which contained 20 ng template and a final primer concentration of 0.6 µM. 18 s rRNA (18 s Ribosome RNA) was used as internal reference genes for semiquantitative analysis.

Independent Samples T Test (SPSS Version 16.0) was used to evaluate differences of mRNA expression between groups of UM cells and NUM. The difference was defined as significant at p<0.05 and highly significant at p<0.01.

## Results

### Characterization of the two cDNA libraries

In order to obtain a thorough survey of the transcriptome of UM, we used NUM as the controls and sequenced both of them to a depth of more than 50 million reads for analysis (**Table 1**). A total of 84,591,386 and 47,744,976 tags were sequenced separately via the strategy of filtering low-quality reads. After discarding remnant rRNA from the two libraries, we mapped these tags (∼35 bp in length) to the human genome and a database of unique exon-junction sequences, using the ABI SOLiD sequencing system. We adopted the dual-matching strategy to ensure capturing of both exonic expression and combinatorial exon usage for all loci. To maximize tag-mapping accuracy and coverage, we made triplicate-alignment strategy and only used high-quality sequences of at least 25 nt. In the library of UM, 37,964,619 tags in all were mapped to specific locus of chromosome or exon-junction database, of which 33,238,428 were focused on multiple regions and 4,726,191 were on unique region. In comparison of NUM library, 27,011,204 tags were mapped to specific locus of chromosome or exon-junction database, including 6,102,251 unique mapping regions and 20,908,953 multiple mapping regions. Both of the mapping rates of the two libraries were almost 50%.

**Table 1:** Tag mapping summary.

Read mapping	UM	NUM
Raw tag number	92,718,536	55,464,728
High-quality reads	84,591,386	47,744,976
35 bp mapped reads	13,042,363	10,522,399
30 bp mapped reads	12,925,983	9,316,993
25 bp mapped reads	11,996,273	7,171,812
Reads Mapped to unique locus	4,726,191	6,102,251
Reads Mapped to multiple loci	33,238,428	20,908,953
Annotated tag number	37,964,619	27,011,204
Annotation rate	44.88%	56.57%
Unique reads mapped to exon	3,608,762	5,099,313
Unique reads mapped to intron	759,012	398,644

Tags mapping to unique locus were used to analyze the distribution of transcripts. Based on Ensembl dataset, we found that 76.36%–83.56% transcripts were located from exon region and 16.06%–6.53% transcripts were from intron region in two libraries. Meanwhile, the residual of over 6% tags were from gene region of anti-sense strand (**Table 1**).

### Gene profiling and the category of highly-expressed genes in two libraries

After mapping triplicate-alignment strategy, 19,515 and 19,714 genes were annotated with detailed information. To avoid the influence of background noise and sequencing errors, genes with more than one tag annotated on exon regions were considered expressed. According to strict statistical strategy of annotation, we confirmed 14,201 and 14,236 expressed genes in two libraries, respectively. From the most abundant genes, we discovered that most of them were related to melanocyte pathphysiological changes relevant to metabolisms of cell cycle and apoptosis (**Table S3**), which were associated with these two cell types.

### Functional distribution of differentially-expressed genes

Up- and down-regulated genes were considered to be essential determinants of tumor development. To better survey the biological behavior of tumor growth and progression, it is necessary to familiarize with the functional distribution of these differentially-expressed genes in malignancies, compared to the normal tissue.

Proceeding normalization of the two libraries by DEGseq program, uniquely mapped reads with one gene annotation in genome were used to identify corresponding RPKM of the gene. Based on the values of RPKM, 9,335 differentially expressed genes with p<0.01 were found between the two libraries. Though the number of expressed genes in two libraries was nearly the same, 5,155 genes (with p<0.01 and fold change value>2) were found to have extremely distinct expression, in which 3,802 genes were up-regulated and 1,353 down-regulated in UM cells compared to NUM.

**Figure 1:**
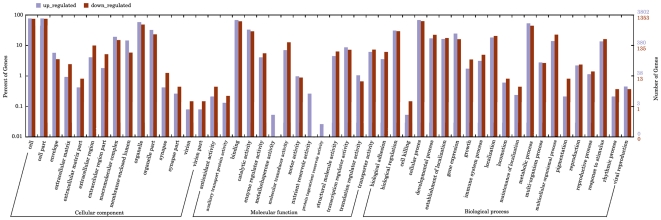
Functional categorization of differentially expressed genes based on known genes in the Uniprot database. Gene Ontology (GO) terms at the 2nd level were plotted here, and in this ontology, “Cellular component”, “Molecular function”, and “Biological process” are categorized independently. The purple and red pillars represent the up-regulated genes and down-regulated genes, separately.

Based on the analysis of GO categories, these 5,155 differentially-expressed genes were categorized into three major functional groups: cellular component, molecular function, and biological process. The abundant genes were categorized into 17 major functional groups (Percentage of expressed genes>10) based on the GO categories after exclusion of crystallin, rRNA, and mitochondrial genes. The top six functional categories include metabolic process, cellular process, cell, organelle, binding, and catalytic activity (**Figure 1**). The third level GO categories were consistent with these results and provided more information on cellular functions and subcellular locations. The major functional groups based on these categories included intracellular, membrane-bound organelle, ion binding, hydrolase activity, nucleic binding, protein binding, metabolism of localization, macromolecule metabolic localization, primary metabolism process, translator cell communication, regulation of cellular process, and organismal physiologic process. Furthermore, it has been found that major functional categories of up-regulated genes in UM included metallochaperone activity, nutrient reservoir activity, and protein tag, which indicated that occurrences of UM or metastatic propensity is correlated with increased synthesis of certain proteins.

### Expression coverage of susceptive chromosome

**Figure 2:**
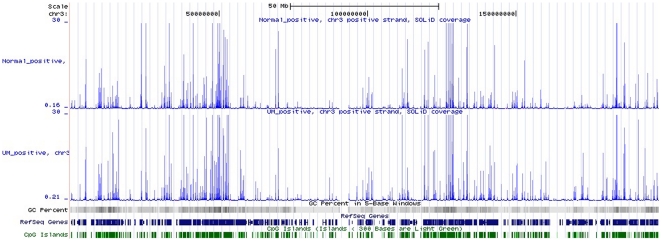
Distributions of differentially expressed genes in chromosome 3 compared with RefSeq Genes, GC content, and CpG Islands.

Chromosome 3 deletion is found in several cancers, monosomy 3 is the most common abnormality of chromosomes in the pathogenesis of UM, which has been found to be associated with UM metastasis. However, only a few reports described the loss of heterozygosity of this chromosome. We could not find extreme deletion of chromosome 3, while the expression values of UM in chromosome 3 were lower than NUM (**Figure 2**). Meanwhile, the UM expressed region had a range wider than NUM, which reflected that the mutation rate may be higher than normal cells (**Table 2**).

**Table 2:** Tag expression information of Chromosome 3 between UM and NUM.

Sample	UM	NUM
Start	123964	98702
End	197900737	197810160
Expression values	778303.04	599828.29
Bases covered	969,900 (0.49%)	746,095 (0.38%)
Minumum value	0.21	0.16
Maximum value	157	105.21
Range	156.79	105.05
Mean value	0.802457	0.803957
Variance	6.18828	6.6656
Standard deviation	2.48763	2.58178
Database	GRCh37/hg19	GRCh37/hg19

**Figure 3:**
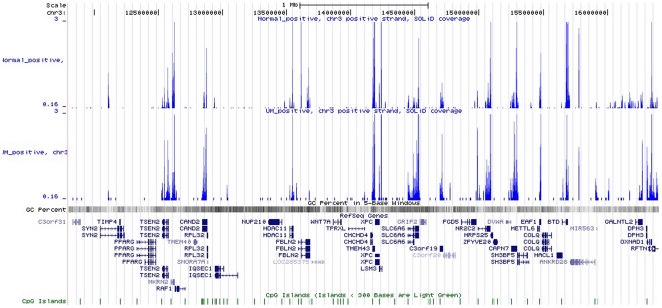
Distributions of differentially expressed genes in the region of 3p25.1 to 3p25.2 in chromosome 3.

Previous atudies have reported interstitial or translocation of certain regions (mainly 3p25.1 to 3p25.2) in chromosome 3 in UM. We used transcripts annotated between 3p25.1 to 3p25.2 to analyze the expression difference in this region by UCSC (**Figure 3**). According to NCBI annotation, 43 genes are mapped to the region (Build 34 version 1, found at NCBI). From **Figure 3**, we found that the importance of this region was due to the expression variation of corresponding oncogenes and tumor suppressor genes.

## Discussion

### Variation of biological pathways related to UM

The transferred process from NUM to malignant posterior UM is not induced by one metabolism pathway, but as a result of alterations of multiple biological pathways that are from the mutation of multiple oncogenes and turmor suppressor genes. During the metastatic process of melanoma, involved pathways are mainly composed of the following: MAPK signaling pathway, cell cycle, p53 signaling pathway, apoptosis, and melanogenesis. According to strict selection, we used these 5,155 genes with extreme remarkable differentiation and uniquely described information to annotate the relative protein or transcript of these pathways, which was expected to describe the variation of gene expression in relative biological pathways.

**Figure 4:**
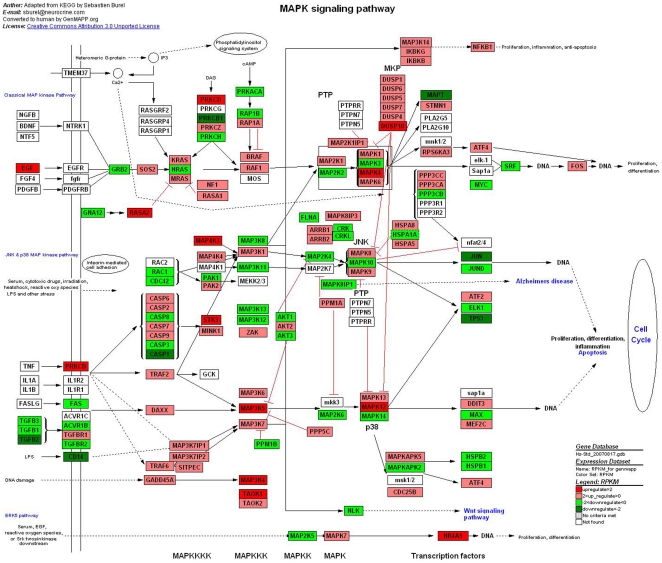
Different genes expression in MAPK signaling pathway. Red and green represent up-regulated and down-regulated genes, individually. The regulation extents are based on the color grade.

MAPK signaling pathway is a basic signal transduction pathway that couples intracellular responses to the binding of growth factors to cell surface receptors, which regulates the cell proliferation, survival, differentiation and apoptosis [38]. MAPK signaling pathway is composed of three different major signaling pathway—ERK, JNK and p38. GNAQ mutation is detected in half of UM clinical cases but has not been found in cutaneous melanomas [39]. This mutation induces activation of one of the MAPK signal pathways (ERK1/2) and upregulation of PKA [40]. In our analysis (**Figure 4**), we found that RAS up-regulation activated the expression of ERK by multiplex phosphorylation, which finally regulate the mitosis by activating MARK. The lack of BRAF mutations in posterior UM predicates that missense mutations of BRAF is not related to the unlimited cell proliferation in UM, which is differnt from the pathogenesis of cutaneous melanoma. It may reflect that the pathogenesis of uveal melanoma is not completely identical with cutaneous melanoma.

**Figure 5:**
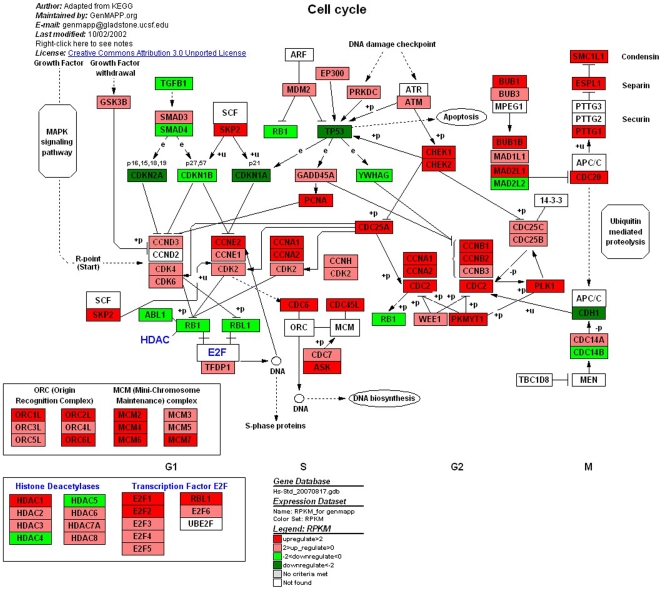
Different genes expression in cell cycle. Red and green represent up-regulated and down-regulated genes, individually. The regulation extents are based on the color grade.

The cell cycle is an ordered, tightly regulated process that takes place in a cell leading to its division and duplication (replication) [41]. Some proteins related to Cyclins, RB, and p53, are checkpoints that control cell cycle progression of UM [20]. Checkpoint loss results in genomic instability and has been implicated in the transformation of normal cells into cancer cells [42]. There were alternation of expression of 103 genes in cell cycle pathway, in which 75 genes were up-regulated, and 28 genes were down-regulated (**Figure 5**). Most of the up-regulated genes were related to DNA synthesis and transcriptional activation in different phases of cell cycle. Furthermore, the expressions of transcriptional inhibitors were evidently reduced, like *cdkn2a, cdkn1a, cdkn1b* and *mad1l2.* We particularly noticed the deregulation of the retinoblastoma gene (*Rb*), whose protein product is the prototype tumor suppressor gene by virtue of its central role in regulating the cell cycle [43]. In most other malignancies, *Rb* is functionally inactivated by inappropriate phosphorylation resulting from deregulation of upstream effectors in the *Rb* pathway (eg, p16 inactivation or cyclin D overexpression) [44]. In our analysis, we concluded that *Rb* may be functionally inactivated in UM as a result of cyclin/cdk complex phosphorylation that blocks its tumor suppressor activity. This would then lead to inactivation and ceaselessness of cell division in UM cells.

**Figure 6:**
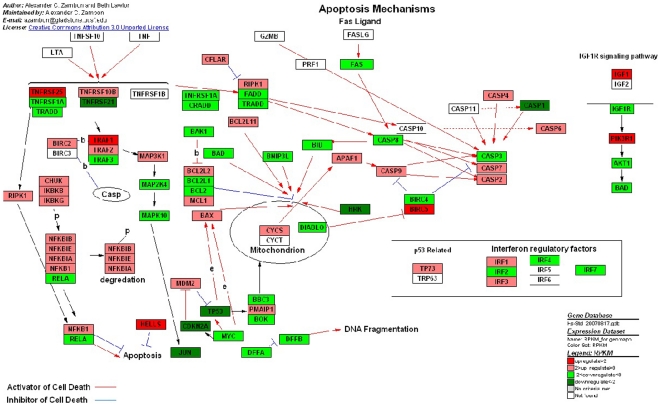
Different genes expression in apoptosis mechanism. Red and green represent up-regulated and down-regulated genes, individually. The regulation extents are based on the color grade.

Apoptosis is an important mechanism for maintaining cellular homeostasis, preventing the accumulation of deleterious mutations, and averting malignant transformation [45]. The process of apoptosis is controlled by a diverse range of cell signals, which may originate either extracellularly (toxins, hormones, growth factors, nitric oxide or cytokines) or intracellularly [46]. However, cancer cells can become resistant to apoptosis by activating proteins that protect the cell from apoptosis, by mutating proteins that cause apoptosis or by developing the ability to ignore apoptotic signals. Apoptosis mechanisms pathway displayed the most of genes expressions changes, which were 35 genes were up-regulated and 33 genes wee down-regulated (**Figure 6**). The initiation of apoptotic mechanisms was induced by the activation of TNF receptor or Fas receptor [47], [48]. The down-expression of these receptors (like TNFRSF1A, TNFRSF2A and TRADD) in UM restricted the combination reaction with other cells that have relative ligands. Furthermore, Fas receptor (also known as *Apo-1* or *CD95*) binds the Fas ligand (FasL), a transmembrane protein part of the TNF family [47]. The interaction between Fas and FasL results in the formation of the *death-inducing signaling complex* (DISC), which contains the FADD, caspase-8 and caspase-10. From the analysis (**Figure 6**), down-regulation of related caspases in UM inhibites the ongoing apoptosis and activates pondent antiapoptosis mechanisms.

**Figure 7:**
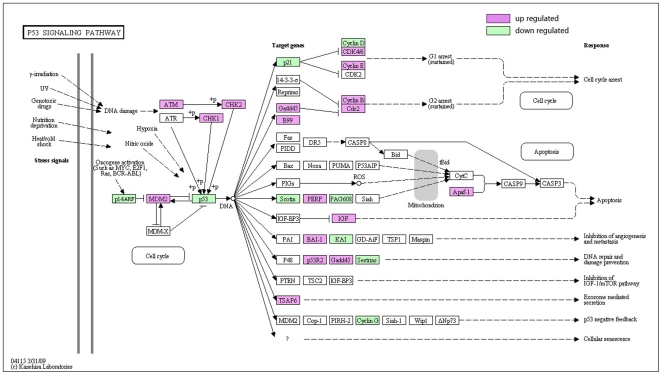
Different genes expression in P53 signaling pathway. Red and green represent up-regulated and down-regulated genes, individually. The regulation extents are based on the color grade.

P53 is a key apoptotic regulator that is mutated in most kinds of human cancer [49], [50], [51]. Its inactivation, induced by a number of stress signals, including DNA damage, oxidative stress and activated oncogenes, always results in three major outputs: cell cycle arrest, cellular senescence or apoptosis [52]. According to our analysis in cell cycle pathway, the inactivation of P53 leads to the continual process of DNA damage cell from G1 to S without proper repair. With the down-regulation of P53 (**Figure 7**), other p53-relatived genes could not communicate with adjacent cells, repair the damaged DNA or set up positive and negative feedback loops that efficiently open up the pathway of apoptosis.

**Figure 8:**
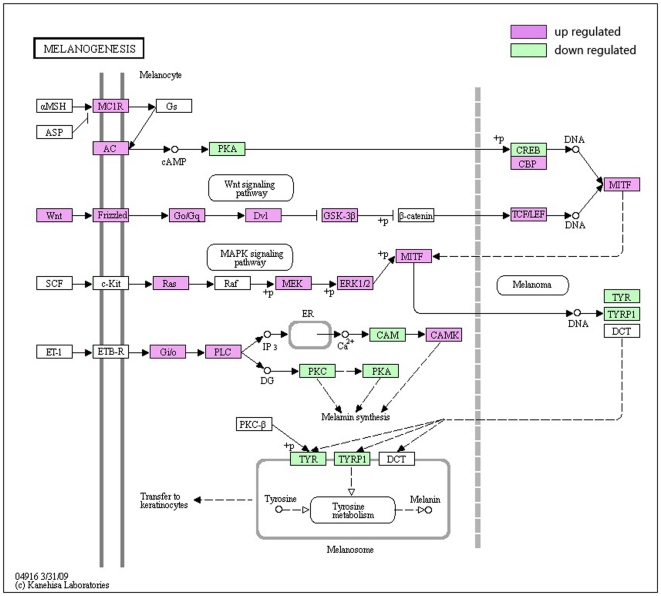
Different genes expression in Melanogenesis metabolism. Red and green represent up-regulated and down-regulated genes, individually. The regulation extents are based on the color grade.

Melanogenesis is under complex regulatory control by multiple factors. The most important positive regulator of melanogenesis is the MC1 receptor with its ligands melanocortic peptides [53]. It has been reported that MC1R was expressed in 95% of UMs and no staining could be found in adjacent normal tissues [54]. MC1R activates the cyclic AMP (cAMP) response-element binding protein (CREB). Increased expression of MITF and its activation by phosphorylation (P) stimulate the high-regulated transcription of tyrosinase (TYR), tyrosinase-related protein 1 (TYRP1), and dopachrome tautomerase (DCT), which is involved in tyrosine metabolism in melanosomes to produce melanin (**Figure 8**). Furhtermore, it has been reported that MC1R induces phosphorization of ERK [55], which may cause unlimited cell proliferation and oncogenesis.

### Quantitative real time PCR expression profiling of selected genes

To prove the authenticity of the SOLiD, the expression levels of selected genes were validated on cultured cell lines from other cell lines of posterior UM and NUM by qRT-PCR. Thirty-one candidate genes (**Table S2**) have been randomly selected from genes that were differentially expressed throughout SOLiD sequencing. We also used one endogenous control 18 rRNA to exclude possible influences of regulation. The qRT-PCR results confirmed that 21 of them showed identical gene expression patterns as what were found via SOLiD sequencing technique (**Table S4**). Eight genes that failed to be validated in qRT-PCR were poorly expressed genes.

Comparing the expression data of RNA-Seq, qRT-PCR was sufficiently sensitive to confirm high expressed genes, and the results were identical with SOLiD data; however, it was not as sensitive in the detection of low-expressed genes, which may lead to the unsuccessful validation of the nine genes. Therefore, we make the conclusion that qRT-PCR is not as sensitive detecting low expressed genes when compared to SOLiD sequencing technology.

### Conclusions

Throughout the present study, we found a large number of potentially interesting genes for biological investigation of UM. The expression profiling suggests that the formation of UM occurs along with the variation of several metabolisms, such as MAPK signal pathway, cell cycle, apoptosis, P53 pathway and melanogenesis. Our study indicates that SOLiD is a useful technique for functional genomic and transcriptome research of various tumor. In the meantime, several potential markers were found to discriminate between between UM and NUM.

## Supporting Information

Table S1 **Summary of library examination between UM and NUM.**
(DOC)

Table S2 **Summary of Quantitative Real-Time PCR primers.**
(DOC)

Table S3 **Gene profiling and the category of highly-expressed genes in two libraries.**
(XLS)

Table S4 **Quantitative real time PCR expression profiling of selected genes.**
(DOC)
