# Supplementary material for: A Comparative Transcriptomic Analysis of Uveal Melanoma and Normal Uveal Melanocyte
Source: PLoS One. 2011 Jan 28;6(1):e16516. doi: 10.1371/journal.pone.0016516 (PMC3030591; doi:10.1371/journal.pone.0016516)
Supplement: Table S1 — Summary of library examination between UM and NUM. (DOC) [file pone.0016516.s001.doc]

**Table S1: Summary of library examination between UM and NUM**

| Tissue Sample  Statistic | UM | NUM |
| --- | --- | --- |
| Length less than 20 bp | 1 | 2 |
| Length between 20bp and 40 bp | 1 | 3 |
| Length more than 40 bp | 27 | 34 |
| Total examined reads | 29 | 39 |
|  |  |  |
| Reads mapped to mRNA | 26 | 39 |
| Reads mapped to rRNA | 0 | 0 |
| Reads mapped to non-coding RNA | 1 | 0 |
| Unmmapped reads | 2 | 0 |
|  |  |  |
| Solution concentration | 34.7ng/μl | 69.4ng/μl |
| Solution volume | 10μl | 10μl |
