# Supplementary material for: A Comparative Transcriptomic Analysis of Uveal Melanoma and Normal Uveal Melanocyte
Source: PLoS One. 2011 Jan 28;6(1):e16516. doi: 10.1371/journal.pone.0016516 (PMC3030591; doi:10.1371/journal.pone.0016516)
Supplement: Table S2 — Summary of Quantitative Real-Time PCR primers. (DOC) [file pone.0016516.s002.doc]

**Table S2: Summary of Quantitative Real-Time PCR primers**

| Gene Name | NCBI NO. | Positive-strand (5'-3') | Negative-strand (3'-5') | length(bp) |
| --- | --- | --- | --- | --- |
| IGF1 | NM 00618.3 | GTCCTCCTCGCATCTCTTCTACC | CCGACTGCTGGAGCCATACC | 172 |
| E2F1 | NM 005225.2 | GCCAAGAAGTCCAAGAACCACAT | GCTGCTGCTCGCTCTCCTG | 118 |
| PIK3R | NM 181504.2 | CTGTAGTGGTGGACGGCGAA | AGGGAGGTGTGTTGGTAATGTAGC | 131 |
| CDK6 | NM 001259.6 | TGCCGCTCTCCACCATCC | GCACACATCAAACAACCTGACC | 89 |
| CDH1 | NM 004360.3 | ATTCTGATTCTGCTGCTCTTGCT | CCTCTTCTCCGCCTCCTTCTT | 128 |
| TP53 | NM 000546.4 | TGCGTGTGGAGTATTTGGATGA | AGTGTGATGATGGTGAGGATGG | 169 |
| CDKN2B | NM 078487.2 | CGCCCAACTCCACCAGATA | ACTCCACTCCACCACCTCATC | 189 |
| COX4I1 | NM 001861.2 | GCGTGACCACCCCTTGCC | GCCTTCTCCTTCTCCTTCAATG | 81 |
| MT2A | NM 005953.3 | CGCTCCCAGATGTAAAGAACG | TAGCAAACGGTCACGGTCAG | 76 |
| SGK1 | NM 005627.3 | ACAACAGCACAACATCCACCTTC | GCACCACCAGTCCACAGTCC | 98 |
| CASP8AP2 | NM 001137667.1 | TGGCAGCAGATGATGACAATG | GGTACACAGGATTTGGAAGCG | 152 |
| CCNL1 | NM 020307.2 | AGGTGGCGATGGCAACG | GGCGGAGGTGGTGGAATAC | 173 |
| MAPK1 | NM 002745.2 | GGATGTGGTGTTATGGAAAGAGC | AAGAAAGCAGAGACGCAGAATGAC | 118 |
| NFκB1 | NM 003998.2 | GCCCACTCGCTGCCTCT | GTCTCCACGCCGCTGTC | 92 |
| NRAS | NM 002524.3 | CTACCTCCTCACTTGGCTGTCTG | GTTTGCGGTTTGGTTCTCTGTT | 160 |
| RAD9A | NM 004584.2 | GCTGTTCTGCCCTTCTCTCCT | TCTGCCTCCTCCTCGTGGTA | 98 |
| RTKN | NM 001015056.1 | TCTTGGCTCACACCACACTCAC | GACGGCAACACACGCTACC | 127 |
| E2F4 | NR 026947.1 | GCTCACTCCCACTGCTGTCC | TGCTGCTGCTGCTGCTGT | 197 |
| MALAT1 | NR 002819.2 | GCAGCAGTTCGTGGTGAAGATAG | CGCCTCCTCCGTGTGGTT | 190 |
| TRIM28 | NM 005762.2 | GTGTGCTGGTCAATGATGCC | GTGCTCCTGGTGCTTCTGG | 104 |
| CDKN1A | NM 000389.3 | CCAGCGACCTTCCTCATCCA | CCATAGCCTCTACTGCCACCAT | 191 |
| CDKN2A | NM 000077.3 | GGGGTCGGGTAGAGGAGGT | ACGGGTCGGGTGAGAGTG | 180 |
| GAPDH | NM 002046.3 | GCTCTCTGCTCCTCCTGTTC | GACTCCGACCTTCACCTTCC | 99 |
| PTPRS | NM 130855.2 | GCGTCATTGAGGCGGTTG | TGGTGATGCTGGTGGCTGT | 95 |
| ATM | XM 940791.2 | TGCCAGACAGCCGTGACTTAC | ACCTCCACCTGCTCATACACAAG | 97 |
| CREB3 | NM 006368.4 | GTCTCCTTCTGCCTCCTCCTT | GTCTGAGCCGTCCAACCACT | 191 |
| KIT | NM 001093772.1 | CTGGGATTTTCTCTGCGTTCTG | CTGGATGGATGGATGGTGGA | 103 |
| MCHR1 | NM 005297.3 | GCTGCGGACAAAGAGGGTG | GGGCGGCTGATGGACAAC | 117 |
| RASL10B | NM 033315.3 | AGAGGGAGCGGATGAAGGAT | GTTGAGGTGGAGGCGGAAG | 155 |
| CYB5R2 | NM 016229.3 | GCGGGAGGAGAGGCAACT | TCAGGACCAACACGAGCACA | 127 |
| MT-CO2 | XR 078889.1 | GGCGACCTGCGACTCCT | GTGTAGCGGTGAAAGTGGTTTG | 171 |
| 18s rRNA | NM 003286.1 | TCAACACGGGAAACCTCACC | TCGCTCCACCAACTAAGAACG | 110 |
