# Supplementary material for: A Comparative Transcriptomic Analysis of Uveal Melanoma and Normal Uveal Melanocyte
Source: PLoS One. 2011 Jan 28;6(1):e16516. doi: 10.1371/journal.pone.0016516 (PMC3030591; doi:10.1371/journal.pone.0016516)
Supplement: Table S4 — Quantitative real time PCR expression profiling of selected genes. (DOC) [file pone.0016516.s004.doc]

**Table S4.** Quantitative real time PCR expression profiling of selected genes

|  |  | Normal cells | | | | | | Tumer cells | | | |
| --- | --- | --- | --- | --- | --- | --- | --- | --- | --- | --- | --- |
| Cell Line | | M1 | UM-U90 | UM-U94 | UM-U95 | UM-U97 | UM-U106 | M17 | M21 | M23 | S65 |
|  | RQ |  | 0.86 | 1.59 | 1.70 |  | 0.43 | 0.00 | 0.01 | 0.00 |  |
| CDH1 | mean±SD | 1.14±0.60 | | | | | | 0.002±0.003 | | | |
|  | P Value | 0.02 | | | | | | | | | |
|  | RQ | 0.64 | 0.72 | 2.05 | 1.82 | 1.53 | 0.37 | 13.71 | 24.48 | 32.22 | 11.20 |
| CDK6 | mean±SD | 1.19±0.70 | | | | | | 20.4±9.76 | | | |
|  | P Value | <0.01 | | | | | | | | | |
|  | RQ | 0.59 | 1.52 | 1.44 | 1.56 | 0.72 | 0.68 | 4.50 | 4.09 | 3.89 | 4.31 |
| E2F1 | mean±SD | 1.09±0.47 | | | | | | 4.20±0.26 | | | |
|  | P Value | <0.01 | | | | | | | | | |
|  | RQ | 2.76 |  |  | 2.30 | 0.31 | 0.51 | 1201.40 | 2055.11 | 2304.12 | 1481.15 |
| IGF1 | mean±SD | 1.47±1.24 | | | | | | 1760±507.59 | | | |
|  | P Value | <0.01 | | | | | | | | | |
|  | RQ | 0.96 | 0.92 | 1.85 | 3.12 | 0.79 | 0.25 | 51.32 | 93.47 | 57.62 | 26.88 |
| PIK3R1 | mean±SD | 1.31±1.02 | | | | | | 57.32±27.51 | | | |
|  | P Value | <0.01 | | | | | | | | | |
|  | RQ | 0.63 | 0.64 | 1.20 | 1.15 | 1.84 | 0.97 | 0.35 | 0.46 | 0.45 | 0.52 |
| TP53 | mean±SD | 1.07±0.45 | | | | | | 0.45±0.07 | | | |
|  | P Value | 0.025 | | | | | | | | | |
|  | RQ |  | 1.38 | 6.94 | 0.24 | 0.49 | 0.88 |  |  |  |  |
| CDKN2B | mean±SD | 1.99±2.80 | | | | | | no detection | | | |
|  | P Value |  | | | | | | | | | |
|  | RQ |  | 0.48 | 9.15 | 0.17 | 2.18 | 0.60 | 276.95 | 408.73 | 494.56 | 464.65 |
| COX411 | mean±SD | 2.52± 3.79 | | | | | | 411.22±96.32 | | | |
|  | P Value | <0.01 | | | | | | | | | |
|  | RQ |  | 0.49 | 1.25 | 0.34 | 3.19 | 1.49 | 836.37 | 459.68 | 1158.87 | 384.28 |
| MT2A | mean±SD | 1.35±1.14 | | | | | | 709.80±358.80 | | | |
|  | P Value | 0.029 | | | | | | | | | |
|  | RQ |  | 0.22 | 5.55 | 0.40 | 1.87 | 1.10 | 235.89 | 268.45 |  | 323.70 |
| SGK1 | mean±SD | 1.83±2.18 | | | | | | 276.01±44.39 | | | |
|  | P Value | <0.01 | | | | | | | | | |
|  | RQ |  |  |  |  |  |  | 0.45 | 1.00 | 1.80 | 1.22 |
| CASP8AP2 | mean±SD | no detection | | | | | | 1.12±0.56 | | | |
|  | P Value | <0.01 | | | | | | | | | |
|  | RQ |  | 0.13 | 8.01 | 0.27 | 1.96 | 1.85 | 624.69 | 879.48 |  | 858.10 |
| CCNL1 | mean±SD | 2.44±3.23 | | | | | | 787.42±141.33 | | | |
|  | P Value | 0.011 | | | | | | | | | |
|  | RQ |  | 0.29 | 6.31 | 0.25 | 1.45 | 1.55 | 125.06 | 343.34 | 280.33 | 120.22 |
| MAPK1 | mean±SD | 1.97±2.50 | | | | | | 217.24±112.24 | | | |
|  | P Value | 0.031 | | | | | | | | | |
|  | RQ |  | 0.52 | 3.41 | 0.43 | 1.97 | 0.66 | 156.28 | 219.18 | 114.05 | 148.83 |
| NFKB1 | mean±SD | 1.40±1.28 | | | | | | 159.59±43.79 | | | |
|  | P Value | <0.01 | | | | | | | | | |
|  | RQ |  | 0.38 | 7.63 | 0.38 | 1.23 | 0.74 | 4408.28 | 5417.83 | 4311.58 | 2602.17 |
| NRAS | mean±SD | 2.07±3.13 | | | | | | 4184.96±1167.78 | | | |
|  | P Value | <0.01 | | | | | | | | | |
|  | RQ |  | 0.15 | 2.33 |  | 2.41 | 1.24 |  | 458.41 | 909.54 | 1064.53 |
| RAD9A | mean±SD | 1.53±1.07 | | | | | | 810.83±314.89 | | | |
|  | P Value | 0.047 | | | | | | | | | |
|  | RQ |  | 0.08 | 6.17 | 0.25 | 2.94 | 2.78 | 1066.01 | 707.46 | 2077.31 | 2276.34 |
| RTKN | mean±SD | 2.44±2.48 | | | | | | 1531.78±763.42 | | | |
|  | P Value | 0.028 | | | | | | | | | |
|  | RQ |  | 0.31 | 0.99 | 0.70 | 3.98 | 1.17 | 108.50 | 72.96 | 210.62 | 145.61 |
| E2F4 | mean±SD | 1.43±1.46 | | | | | | 134.42±58.83 | | | |
|  | P Value | 0.02 | | | | | | | | | |
|  | RQ |  | 0.88 | 1.84 | 0.77 | 0.8 |  | 179920.49 | 172770.81 | 174334.7 | 148695.4 |
| MALAT1 | mean±SD | 1.07±0.52 | | | | | | 168930.33±13834.67 | | | |
|  | P Value | <0.01 | | | | | | | | | |
|  | RQ |  | 0.43 | 4.6 | 0.22 | 1.36 | 1.7 | 6.02 | 21.38 | 34.02 | 18.03 |
| TRIM28 | mean±SD | 1.66±1.75 | | | | | | 19.86±11.52 | | | |
|  | P Value | <0.01 | | | | | | | | | |
|  | RQ |  | 0.87 | 1.18 | 0.92 | 1.91 | 0.52 | 6.72 | 7.71 | 4.12 | 2.66 |
| GAPDH | mean±SD | 1.08±0.52 | | | | | | 5.30±2.32 | | | |
|  | P Value | 0.033 | | | | | | | | | |
